# MicroRNA-2187 Modulates the NF-κB and IRF3 Pathway in Teleost Fish by Targeting TRAF6

**DOI:** 10.3389/fimmu.2021.647202

**Published:** 2021-02-15

**Authors:** Wenya Gao, Renjie Chang, Yuena Sun, Tianjun Xu

**Affiliations:** ^1^Laboratory of Fish Molecular Immunology, College of Fisheries and Life Science, Shanghai Ocean University, Shanghai, China; ^2^Laboratory of Marine Biology and Biotechnology, Qingdao National Laboratory for Marine Science and Technology, Qingdao, China; ^3^Key Laboratory of Exploration and Utilization of Aquatic Genetic Resources (Shanghai Ocean University), Ministry of Education, Shanghai, China; ^4^National Pathogen Collection Center for Aquatic Animals, Shanghai Ocean University, Shanghai, China

**Keywords:** miR-2187, TRAF6, immune response, negative regulation, fish

## Abstract

The innate immune organs and cells detect the invasion of pathogenic microorganisms, which trigger the innate immune response. A proper immune response can protect the organisms from pathogen invasion. However, excessive immunity can destroy immune homeostasis, leading to uncontrolled inflammation or pathogen transmission. Evidence shows that the miRNA-mediated immune regulatory network in mammals has had a significant impact, but the antibacterial and antiviral responses involved in miRNAs need to be further studied in lower vertebrates. Here, we report that miR-2187 as a negative regulator playing a critical role in the antiviral and antibacterial response of miiuy croaker. We find that pathogens such as *Vibrio anguillarum* and *Siniperca chuatsi* rhabdoviru*s* (SCRV) can up-regulate the expression of miR-2187. Elevated miR-2187 is capable of reducing the production of inflammatory factors and antiviral genes by targeting TRAF6, thereby avoiding excessive inflammatory response. Furthermore, we proved that miR-2187 modulates innate immunity through TRAF6-mediated NF-κB and IRF3 signaling pathways. The above results indicate that miR-2187 acts as an immune inhibitor involved in host antibacterial and antiviral responses, thus enriching the immune regulatory network of the interaction between host and pathogen in lower vertebrates.

## Introduction

Innate immunity and acquired immunity are important ways for the body to protect itself from pathogenic microorganisms (1). Invading pathogens are effectively identified by various extracellular or intracellular pattern-recognition receptors (PRRs) which can recognize conserved signature molecular structures termed as pathogen-associated molecular patterns (PAMPs) (2). PRRs rapidly initiate a series of immune responses by inducing the production of inflammatory cytokines, chemokines, and type I interferon (IFNs) after ligand binding (3). PRRs are a kind of evolutionarily conserved host sensor, including Toll-like receptors (TLRs), RIG-I-like receptors (RLRs), NOD-like receptors (NLRs), and C-type lectin receptors. Among them, TLRs and RLRs are the most studied receptors in the immune responses (4, 5).

TLRs are recognized as the main sensors of pathogens involved in the regulation of innate and adaptive immune system. Once TLRs recognize the molecular structure of pathogens, TLRs will activate the NF-κB transcription factor through activating the myeloid differentiation factor 88 (MyD88) and tumor necrosis factor receptor related factor 6 (TRAF6). Upon TRAF6 activated, it could co-catalyze the synthesis of Lys63-linked polyubiquitin chains with a dimeric ubiquitin conjugating enzyme complex Ubc13-Uev1A thereby activating the protein kinase complex containing TAK1, TAB1, and TAB2, leading to activation and nuclear translocation of transcription factor NF-κB (6, 7). Then, such the inflammatory cytokines including IL-1β, IL-8, and TNF-α were transcribed subsequently. And at the same time, IRF family is also activated by IKK kinase family. Unlike TLRs-mediated antiviral response, RLRs are used as sensors of cytoplasmic to detect viruses. After effective identification of virus intrusion, both RIG-I and MDA5 will interact with the CARD-containing mitochondrial antiviral signal protein (MAVS) which contains multiple TRAF-interacting motifs (TIMs) and can then interact with TRAF family members to signal the transcription of type I IFN and inflammatory cytokines (8). Similar to TLR signaling, TRAF6 also plays an indispensable role in RLR-mediated NF-κB signaling pathway and IRF3 is directly activated by TBK1. It is worth noting that NF-κB activation is an important common event in both TLR and RLR signaling pathways.

TRAF6, an important intracellular multifunctional signaling molecule, was the most widely studied member of TRAF family. TRAF6 contains a highly conserved C-terminal TRAF domain and a helical coiled structure N-terminal activation domain (9). Presently, accumulating evidences have reported that TRAF6 could be activated under VSV infection, bacterial lipopolysaccharide (LPS), and poly(I:C) stimulation (10, 11), which indicated that TRAF6 is critical for both antibacterial and antiviral innate immunity. TRAF6 not only acts as a signal transducer in the NF-κB pathway but activates inhibitor of IκB kinase (IKK) in response to pro-inflammatory cytokines (12). Besides, TRAF6 acts as an E3 ubiquitin ligase to catalyze the K63 polyubiquitination of TAK1, which in turn leads to the activation of IKK and activates downstream signaling molecules, thereby inducing the activation of IRF3, IRF7, and NF-κB (13). The latest report demonstrated that the deletion of TRAF6 promoted the replication of Newcastle disease virus in mouse embryonic fibroblasts (MEF cells) (14). With the increasing importance of TRAF6 in immune regulation, its role in fish has gradually received attention. However, the functions and mechanisms of TRAF6 in fish are poorly understood when compared with mammalian TRAF6.

MicroRNAs are non-coding RNAs consisting of 19–23 nucleotides, which can regulate gene expression by binding to the 3′untranslated region (3′-UTR) of target genes. miRNAs can regulate mRNA expression at the post-transcriptional level mainly by inhibiting the translation of mRNA or promoting the degradation of mRNA (15, 16). According to reports, more than 60% of mRNA is regulated by miRNAs to some extent (17), so miRNAs have become an important transcription factor that controls cell protein content. At present, a number of studies on miRNAs extensively involved in the regulation of TLR and RIG-I pathways at different levels in response to pathogen invasion have been reported (18). For example, the adaptor protein MyD88 associated with TLR has been studied, which can be regulated by miR-3570 and miR-214, thereby regulating the innate immune response (19, 20). And the RIG-I-induced cytokines and antiviral genes also could be strictly constrained by miiuy croaker miR-3570 through targeting MAVS to avoid excess immunity (21). miR-146a, a critical miRNA, inhibits the TLR signaling pathway by targeting IRAK1 and TRAF6 (22). Miiuy croaker miR-203 and miR-21 have been studied in regulating the innate immune response of bacterial infections by inhibiting IRAK4-mediated NF-κB signaling (23, 24). Additionally, a recent study showed that the signaling molecule TAK1 has been regulated by miR-217, and miR-217 also plays important roles in regulating TAK1-induced NF-κB and IRF3 signals (25). Overall, these studies show that miRNAs act as an essential fine-tuning regulator in fish PRRs signaling transduction.

Miiuy croaker (*Miichthys miiuy*) has important medicinal value and is an important economic marine fish. Presently, this species has been used as a model for studying the fish immunology, because extensive researches on its transcriptome, whole genome, functional genes and immune pathway regulation have been reported (26–28). Due to the diversity of the water environment, fish is constantly threatened by bacteria and viruses, among which Gram-negative bacteria and rhabdovirus are important groups. *V. anguillarum* is a Gram-negative pathogen, while SCRV is a member of rhabdovirus, a kind of fish RNA virus. Both of these pathogens can cause severe hemorrhagic septicemia according to reports (29). Therefore, the regulation mechanism of *V. anguillarum* and SCRV infection in teleost is the focus of our research.

In the present study, we report a new regulatory mechanism of miRNA in response to innate immunity. We have explored the expression of miR-2187 and the relationship between miR-2187 and TRAF6 under the stimulation of Gram-negative bacteria or *Siniperca chuatsi* rhabdovirus (SCRV), a typical fish RNA rhabdovirus. Importantly, we found that miiuy croaker miR-2187 could be rapidly upregulated after *V. anguillarum*, LPS, SCRV, and poly(I:C) treatment. Further analysis showed that up-regulated miR-2187 can inhibit the expression of TARF6, and inhibit inflammatory cytokine genes and IFN-stimulating genes (ISG) through TRAF6-mediated NF-κB and IRF3 signaling pathways, thereby avoiding excessive inflammation. This tight negative regulation mechanism plays an important role in attenuating inflammatory response and avoiding excessive inflammation. These data not only provide more theoretical basis for studying miRNA as a negative feedback regulator involved in the antibacterial and antiviral immune response in fish, but enriched miRNA-mediated networks of host-pathogen interactions.

## Materials and Methods

### Sample and Challenge

Healthy Miiuy croakers (weight, ~50 g) come from the Fisheries Research Institute of Zhoushan, Zhejiang Province, China. Before the experiment, the fish was conditioned for at least 6 weeks in an air-filled seawater tanks at 25°C. Bacterial and SCRV challenge were performed as described previously (25, 30). In short, healthy miiuy croakers were challenge with 100 μl of *V. anguillarum* (1.5 × 10^8^ CFU/ml), LPS (InvivoGen, 1mg/ml), poly(I:C) (InvivoGen, 1mg/ml), or SCRV at a multiplicity of infection (MOI) of 5 through intraperitoneal route, and individual challenged with 100 μl of physiological saline as a comparison group. After that, the fish were killed at different time points and the spleen tissues were collected for RNA extraction. All animal experimental procedures were carried out in accordance with the National Institutes of Health's Guide for the Care and Use of Laboratory Animals, and the experimental protocols were approved by the Research Ethics Committee of Shanghai Ocean University.

### Cell Culture and Transfection

Epithelioma papulosum cyprinid (EPC) cells were cultured in medium 199 (Invitrogen) supplemented containing 10% FBS, 1% Penicillin-Streptomycin Solution (100×) under condition with 5% CO_2_ at 26°C. Cells with no stimulation were collected as the control, and each experiment have three biological replicates. Miiuy croaker macrophages were aseptically isolated from the head kidney samples as described (30). The cells were cultured in L-15 (Hyclone) medium supplemented with 15% FBS (Life Technologies) and 1% Penicillin-Streptomycin Solution (100×). Miiuy croaker kidney cell lines (MKC) were cultured in incubator at 26°C. Cells were divided into 24-well or 48-well plates before they were transferred until 80% of cell density.

Prior to transient transfection, cells were seeded into each well of a 24-well or 48-well plate and incubated overnight. Subsequently, EPC cells were transfected with the plasmid using X-tremeGENE HP DNA Transfection Reagent (Roche) according to the manufacturer's protocol. RNA oligoribonucleotides were transfected into MKC cells by using Lipofectamine RNAiMAX (Invitrogen). Washing the macrophages and infecting them with LPS, poly(I:C) or SCRV with MOI of 5, and incubate at different times as indicated.

### Plasmid Construction

In order to construct the TRAF6 expression plasmid, the full-length coding sequence (CDS) region and 3′-untranslated regions (3′UTR) of the miiuy croaker TRAF6 gene were amplified by specific primer pairs and restricted endonuclease sites *Hind* III and *EcoR* I, and then inserted into pcDNA3.1 vector (Invitrogen) with a Flag tag. To construct a TRAF6 3′-UTR plasmid, the full-length TRAF6 3′-UTR region of *M. miiuy, L. crocea*, or *S. ocellatus* were cloned into pmir-GLO luciferase reporter vector to construct the wild type TRAF6-3′UTR plasmid. The mutant-types of the TRAF6 3′-UTR reporter vector were conducted by using Mut Express II Fast Mutagenesis Kit V2 (Vazyme) with mutant primers. Additionally, the wild type of miiuy croaker TRAF6 3′-UTR or the mutant-type was cloned into the mVenus-C1 vector (Invitrogen) which contained the sequence of enhanced GFP. In addition, to construct the pre-miRNA vector, the pre-miR-2187 sequences were amplified by PCR and then cloned into pcDNA3.1 vector (Invitrogen). Correct construction of the plasmids was verified by Sanger sequencing and extracted using endotoxin-free plasmid DNA miniprep kit (Tiangen), before transient transfection, and the expression of protein was confirmed by Western blot analysis. The sequences of all primers are listed in Supplementary Table 1.

### miR-2187 Target Prediction

We used two calculation methods with TargetScan (31), miRanda (32) to predict the targets of miR-2187. Predictions were ranked based on the predicted efficacy of targeting as calculated using the context and scores of the sites.

### Mimics and Inhibitors

miR-2187 mimics (dsRNA oligonucleotides), and control oligo nucleotides were commercially synthesized by GenePharma (Shanghai, China). Their sequences are as follows: miR-2187 mimics were 5′-UUACAGGCUAUGCUAAUCUGU-3′(sense), 5′-AGAUUAGCAUAGCCUGUAAUU-3′(antisense); negative control mimics were 5′-UUCUCCGAACGUGUCACGUTT-3′(sense), 5′-ACGUGACACGUUCGGAGAATT-3′ (antisense); miR-2187 inhibitor (ssRNA oligonucleotides chemically modified by 2′-Ome) were 5′-ACAGAUUAGCAUAGCCUGUAA-3′; inhibitors control were 5′-CAGUACUUUUGUGUAGUACAA-3′.

### RNA Interference

The TRAF6-specific siRNA (si-TRAF6) were 5′-GUGUCACGUAUCUUCAUTT-3′ (sense), 5′-GAUGAAGAUACCGUGACACTT-3′ (antisense). The scrambled control RNA sequences were 5′-UUCUCCGAACGUGUCACGUTT-3′ (sense) and 5′-ACGUGACACGUUCGGAGAATT-3′ (antisense).

### Evaluation of mRNA Levels

Extract viral RNA from intracellular by using the Body Fluid Viral DNA/RNA Miniprep Kit (Axygen). Total RNA which was extracted by TRIzol Reagent (Invitrogen) according to the requirements of manufacturer and cDNA is reverse-transcribed from extracted RNA using the FastQuant RT Kit (Tiangen Biotech), which includes DNase treatment of RNA genome pollution. The expression patterns were performed by using SYBR Premix Ex Taq^TM^ (Takara). Use miRcute miRNA qPCR detection kit for miR-2187 expression analysis (Tiangen). Real-time PCR was performed in an Applied Biosystems® QuantStudio 3 (Thermo Fisher Scientific). β-actin and 5.8S rRNA were employed as internal controls for mRNA and miRNA, respectively as described (33). Primer sequences are displayed in Supplementary Table 1.

### Dual-Luciferase Reporter Assays

For miRNA target identification, EPC cells were co-transfected with wild-type or mutant-type TRAF6 3′-UTR luciferase reporter plasmids, along with miR-2187 mimics, miR-2187 inhibitors, controls or pre-miR-2187 plasmid for 24 h. Additionally, EPC cells were co-transfected with luciferase reporter genes, phRL-TK *Renilla* luciferase plasmid, TRAF6 expression plasmid, along with either miR-2187 mimics or controls. Reporter luciferase activities were measured using the Dual-Luciferase reporter assay system (Promega) (34). After 24 h transfection, they were treated with LPS or poly(I:C) for 6 or 12 h, respectively, then the cells were collected and assayed for reporter activity by using the dual-luciferase reporter assay system. Finally, the relative luciferase activities were determined by calculating the ratio of *Renilla* luciferase activities over firefly luciferase activities. For each experiment, three independent experiments were performed, and each experiment was performed in three times.

### Western Blotting

Total EPC cellular or macrophages lysates were generated by using 1 × SDS-PAGE loading buffer, respectively. Proteins were extracted from cells and were measured with the BCA Protein Assay Kit (Vazyme) and then subjected to SDS-PAGE (10%) gel and transferred to polyvinylidence fluoride (Millipore, USA) membranes by semidry manner (Bio-Rad Trans Blot Turbo System) (35). The membranes were blocked for 1 h with 5% BSA. Then the membranes were incubated at 4°C overnight with anti-flag mouse mAb. Protein was blotted with different antibodies (Abs). The antibody against TRAF6 was diluted at 1: 400 (ProteinTech), anti-Flag and anti-Tubulin monoclonal antibody were diluted at 1:2,000 (Sigma), and HRP-conjugated anti-rabbit IgG or anti-mouse IgG (Abbkine) was diluted at 1:5,000. The results were representative of three independent experiments. The immunoreactive proteins were detected by using WesternBright^TM^ ECL (Advansta). The digital imaging was performed with a cold charge-coupled-device camera.

### Virus Yield Quantification

MKC cells were transfected with RNA oligonucleotides and then infected with SCRV (MOI = 5) as indicated. A volume of 0.1 ml of the cultural supernatant was then serially diluted on the monolayer of EPC cells, and EPC cells were seeded into 96-well plates 1 day before measurement. The 50% tissue culture infectious dose (TCID_50_) was measured after 3 days.

### Statistical Analysis

All experiments were performed with at least three times independently, with three replicates for each experiment. The relative gene expression data was acquired using the 2^−ΔΔ*CT*^ method, and comparisons between groups were analyzed by one-way analysis of variance (ANOVA) followed by Duncan's multiple comparison tests (36, 37). All data are presented as the mean ± SE; a value of *p* < 0.05 was considered significant.

## Results

### RNA Virus and Gram-Negative Bacteria Infection Enhanced miR-2187 Expression

**Figure 1:**
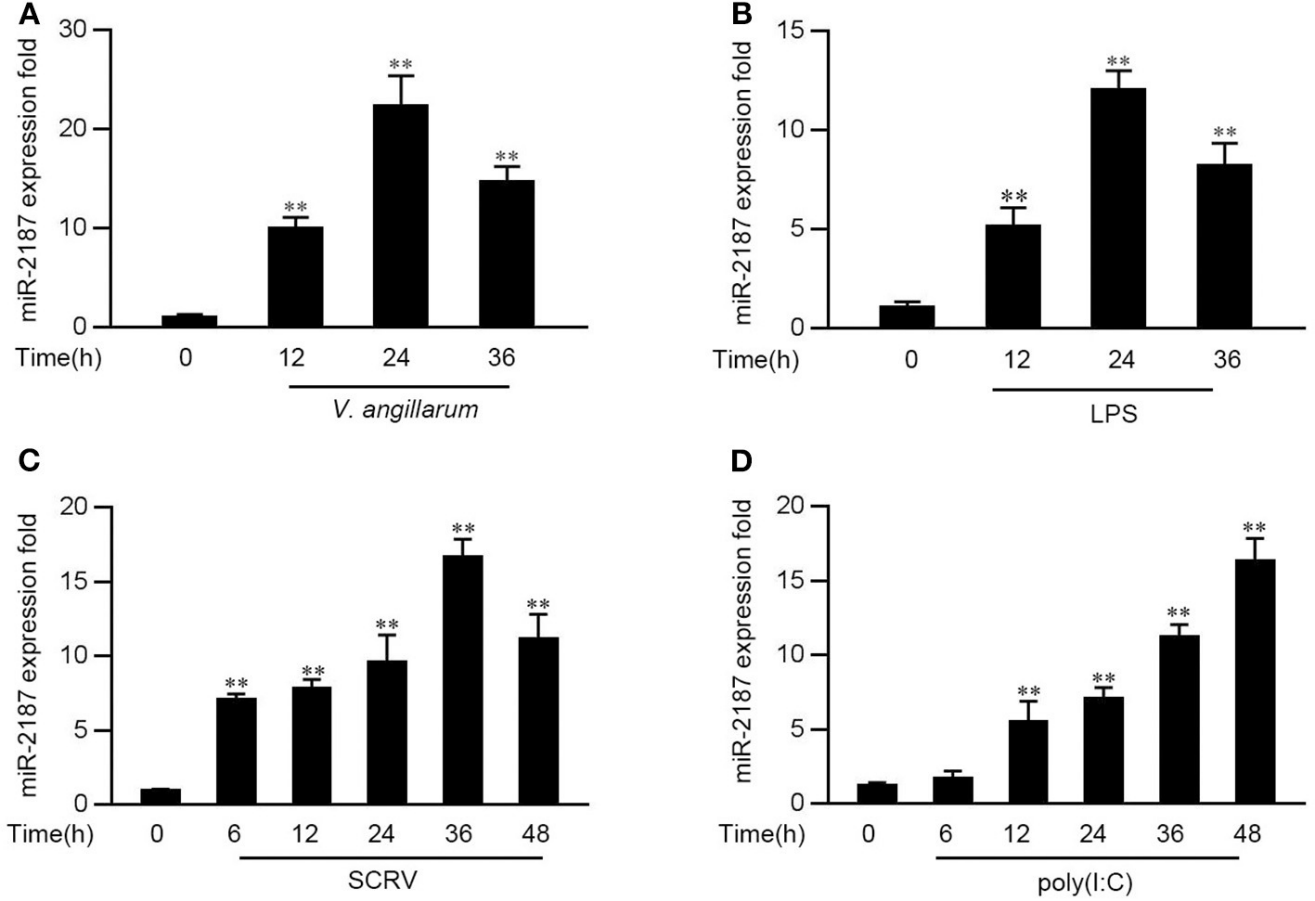
The expression profiles of miR-2187 following Gram-negative bacteria and RNA viral treatment. **(A)** The expression profile of miR-2187 in miiuy croker liver tissues of different time points after infection of the *V. anguillarum* was detected by qRT-PCR. **(B)** The expression profiles of miR-2187 in LPS-stimulated miiuy croaker liver samples. **(C,D)** The expression profiles of miR-2187 in miiuy croaker spleen tissues were measured by qRT-PCR at indicated time after poly(I:C) **(C)** or SCRV **(D)** stimulation. The miR-2187 expression levels were all measured by qRT-PCR and normalized to 5.8S rRNA. Results are standardized to 1 in control samples. All data represented the mean ± SE from three independent triplicated experiments. **p < 0.01 vs. the controls.

In order to explore the influence of pathogen infection on the miRNA profile, the miRNA profile in *V. anguillarum* or LPS-stimulated miiuy croaker liver tissues was investigated firstly. As shown in Figures 1A,B, the level of miR-2187 can be significantly enhanced after the *V. anguillarum* or LPS treatment, and reaches its peak at 24 h, respectively. Furthermore, the expression pattern of miR-2187 was further verified in miiuy croaker spleen tissues following SCRV or poly(I:C) treatment. Similar to the results of LPS stimulation significantly upregulated the expression of miR-2187 at the individual level (Figures 1C,D). These results demonstrated that miR-2187 could be regulated in miiuy croaker in response to Gram-negative bacterial and SCRV infection.

### miR-2187 Depresses the Expression of Inflammatory Cytokines and Antiviral Genes

**Figure 2:**
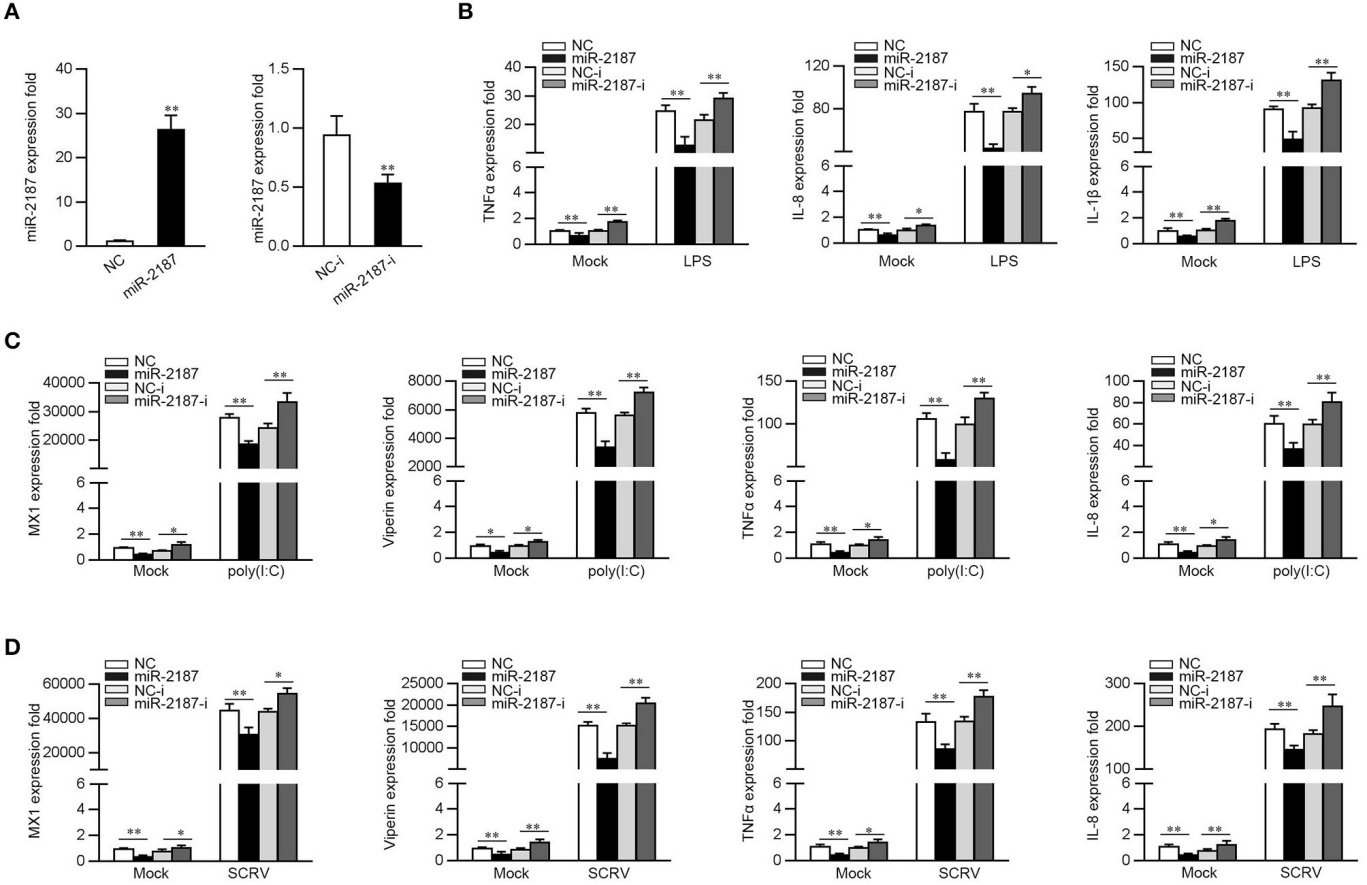
miR-2187 inhibits the expression of inflammatory factors and antiviral genes. **(A)** MKC cells were transfected with control mimics (NC) or miR-2187 mimics (left panel) and negative control mimics (NC-i) or miR-2187 (right panel) for 48 h, the expression of miR-2187 was determined by qRT-PCR and standardized to 5.8S rRNA. **(B)** MKC cells were transfected with NC, miR-2187, NC-i, miR-2187-i for 48 h. Then, the macrophages were treated with LPS for 6 h and the expression of TNF-α, IL-1β, IL-8, and were tested by qRT-PCR and standardized to β-actin. **(C,D)** MKC cells were transfected with NC, miR-2187 mimics, NC-i or miR-2187-i for 48 h, which was stimulated with poly(I:C) or SCRV, and the mRNA level of MX1, Viperin, TNFα, and IL-8 were determined by qRT-PCR normalized to β-actin. All data are presented as the means ± SE from three independent triplicated experiments. **p < 0.01; *p < 0.05 vs. the controls.

Given that miR-2187 can be upregulated upon bacterial and virus infection, then we explored whether miR-2187 could participate in both bacterial- and virus-induced immune response. First, the effect of synthetic miR-2187 mimics and inhibitors on the expression of miR-2187 was assessed in MKC cells. miR-2187 mimics are synthetic double-stranded RNAs (dsRNAs) that simulate naturally occurring mature miRNAs, whereas miR-2187 inhibitors are chemically modified antisense ssRNAs that sequester and inhibit intracellular miRNAs. The results are consistent with expectations, the transfection of miR-2187 mimics sharply enhanced miR-2187 expression whereas miR-2187 inhibitors significantly decreased the expression of miR-2187 (Figure 2A). To confirm the role of miR-2187 in the inflammatory response, we investigated miR-2187 on the regulation of inflammatory cytokine production in LPS-treated macrophages. As shown in Figure 2B, the results implied that the overexpression of miR-2187 mimics could inhibit the expression levels of LPS-induced TNFα, IL-8, and IL-1β. In contrast to the above results, the inhibition of endogenous miR-2187 significantly increased the expression of the indicated inflammatory cytokines compared with the inhibitory control.

To determine whether miR-2187 could regulate inflammatory cytokine and antiviral genes production upon virus infection, the MKC cells were transfected with NC, miR-2187 mimics, NC-i, or miR-2187 inhibitors, then treated with poly(I:C) and the levels of inflammatory cytokines and antiviral genes were tested. As shown in Figure 2C, the expression levels of MX1, Viperin, TNFα, and IL-8 are significantly decreased when MKC were transfected with miR-2187 mimics, while miR-2187 inhibitors upregulated the expression levels of the indicated genes. To further study the function of miR-2187 in regulating RNA virus-induced antiviral immune response, MKC cells were treated with SCRV, then the expression of antiviral genes were detected. As shown in Figure 2D, miR-2187 mimics significantly suppressed the expression levels of MX1, Viperin, TNFα, and IL-8 in SCRV-stimulated MKC cells. Contrary to the above results, miR-2187 inhibitors increased the expression of the corresponding gene. Summarizing the above data shows that miR-2187 acts as a negative regulator to regulate antibacterial and antiviral immune response in miiuy croaker.

### miR-2187 Target TRAF6

**Figure 3:**
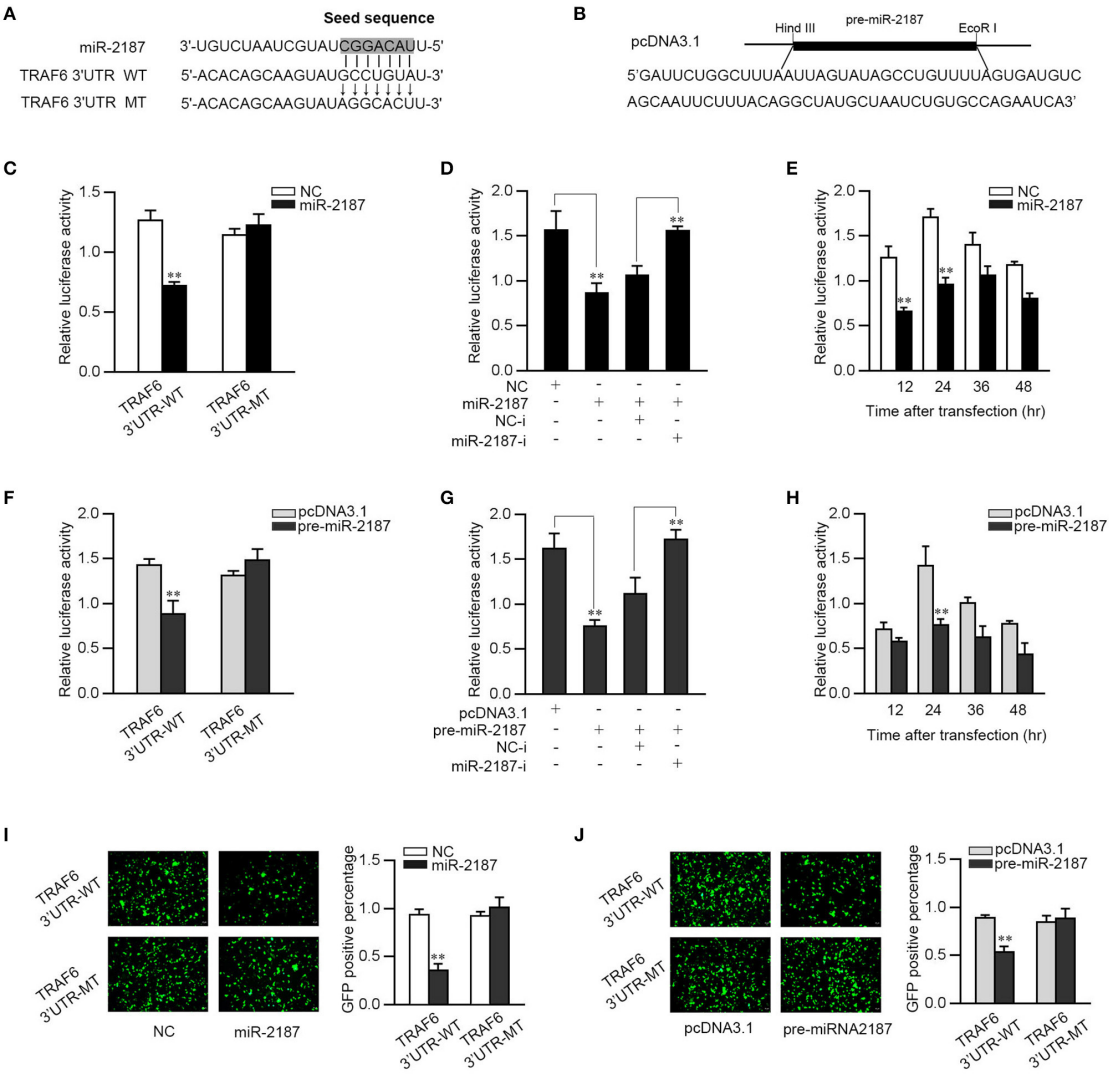
miR-2187 targets miiuy croaker TRAF6 gene. **(A)** Sequence alignment of miR-2187 and its binding site in the 3′-UTR of TRAF6. **(B)** The pre-miR-2187 sequence and the construction of the plasmid. **(C)** EPC cells were transfected with NC or miR-2187 along with wild-type TRAF6-3′UTR (TRAF6-3′UTR-WT) or the mutant type of TRAF6 3′-UTR (TRAF6 3′UTR-MT) for 24 h, the luciferase activity was evaluated. **(D)** EPC cells were co-transfected with TRAF6 3′-UTR-WT, together with NC, miR-2187, NC-i or miR-2187-i 24 h. For each transfection, the total amount of oligonucleotides was controlled and normalized. **(E)** The time gradient experiments were conducted for miR-2187 transfection. **(F)** EPC cells were transfected with pcDNA3.1 or pre-miR-2187 along with TRAF6-3′UTR-WT or the TRAF6 3′-UTR-MT. **(G)** EPC cells were co-transfected with TRAF6 3′-UTR-WT, together with pcDNA3.1, pre-miR-2187, NC-i or miR-2187-i for 24 h. **(H)** The time gradient experiments were conducted for pre-miR-2187 transfection. The wild type or mutant type of TRAF6 3′-UTR was cloned into mVenus-C1 vector. EPC cells were co-transfected with the wild type or mutant type of mVenus-TRAF6 3′-UTR, together with NC or miR-2187 **(I)** and pcDNA3.1 or pre-miR-2187 **(J)**. At 48 h post-transfection, the fluorescence intensity was measured by enzyme-labeled instrument. Scale bar, 20 μm; original magnification × 10. All the luciferase activity was normalized to *Renilla* luciferase activity. All data are presented as the means ± SE from at least three independent triplicated experiments. **p < 0.01 vs. the controls.

In order to identify a possible target, bioinformatics software was used to search for potential miR-2187 targets. After prediction and analysis, we found the 3′-UTR of TRAF6 has a standard sequence for miR-2187 binding. To confirm that TRAF6 is the direct target of miR-2187, TRAF6 3′-UTR luciferase reporter plasmids with the putative target sites for miR-2187 and the mutant type that mutated of miR-2187 targeting sequences were constructed (Figure 3A). Given that miRNA processing system is conserved in vertebrates from mammals to fish, we constructed the pre-miR-2187 sequence plasmid and then transfected it into EPC cells for *in vitro* expression (Figure 3B). Then TRAF6 3′UTR together with miR-2187 mimics or NC were transfected into EPC cells. miR-2187 mimics induces a significant decrease in luciferase activity only in wild-type TRAF6 3′UTR (Figure 3C). Furthermore, as shown in Figure 3D, using miR-2187 mimics and inhibitors to further verify its down-regulation mechanism, and the results revealed that the inhibition of luciferase activity by miR-2187 was weaken after co-transfection with the miR-2187 inhibitors. We also found that the down-regulation mechanism of miR-2187 mimics was time dependent in EPC cells (Figure 3E).

Consistent with the result of miR-2187, when the pre-miR-2187 plasmid and the wild type of TRAF6 3′UTR or the mutant 3′-UTR were co-transfected into the EPC cells, we found that pre-miR-2187 decreased the luciferase activity of the wild-type plasmid but did not reduce the mutant plasmid luciferase activity (Figure 3F). When the luciferase reporter plasmids were co-transfected with the pre-miR-2187 plasmid and miR-2187 inhibitors, we observed that pre-miR-2187 plasmid significantly reduced luciferase activity of the wild-type plasmid, whereas the inhibition effect was weakened by miR-2187 inhibitors (Figure 3G). Pre-miR-2187-triggered time gradient experiment was performed and the result was similar to transfection of miR-2187 (Figure 3H). For further validation, the results revealed that miR-2187 or pre-miR-2187 plasmid could significantly inhibit GFP gene expression, whereas no effect on fluorescence intensity was observed in cells transfected with the mutant form (Figures 3I,J). In summary, these results fully demonstrated that miR-2187 directly targets the 3′UTR of TRAF6.

### miR-2187 Depresses TRAF6 Expression at the Posttranscriptional Level

**Figure 4:**
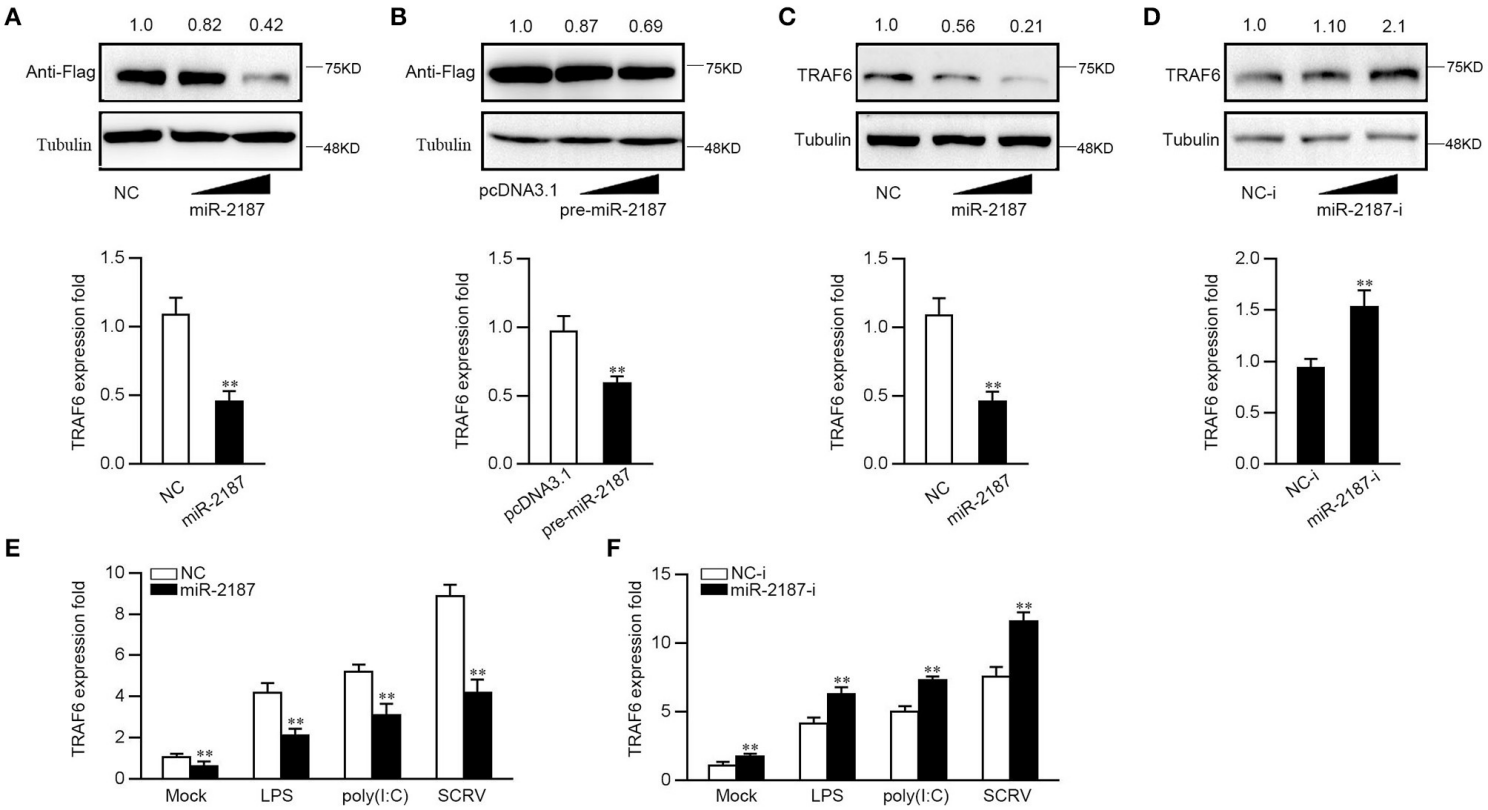
miR-2187 inhibits the expression of TRAF6. **(A,B)** EPC cells were co-transfected with TRAF6 expression plasmid, pcDNA3.1 or pre-miR-2187 along with NC or miR-2187 **(A)** and pcDNA3.1 or pre-miR-2187 **(B)**. After 48 h, western blotting (upper panel) or qRT-PCR (lower panel) was used to determine the expression of TRAF6, respectively. **(C,D)** MKC cells were transfected with NC or miR-2187 **(C)** and NC-i or miR-2187-i **(D)**. After 48 h post-transfection, the protein and mRNA expression of TRAF6 were detected by western blotting (upper panel) or qRT-PCR (lower panel), respectively. **(E,F)** After transfection of NC or miR-2187 **(E)** and NC-i or miR-2187-i **(F)** for 48 h, MKC cells were treated with LPS, poly(I:C) or SCRV, respectively. The mRNA expression of TRAF6 was analyzed by qRT-PCR and normalized to β-actin. All data are presented as the means ± SE from three independent triplicated experiments. **p < 0.01 vs. the controls.

MicroRNAs regulate target genes by binding to their 3′-UTR, and then we next determined whether miR-2187 is involved in the regulation of TRAF6 expression. First, we constructed TRAF6 expression plasmid that includes the full-length CDS region and 3′-UTR of miiuy croaker TRAF6, and then co-transfected with miR-2187 mimics into EPC cells. As shown in Figure 4A, the overexpression of miR-2187 exerted a potent inhibitory effect in dose-dependent manner on the expression of TRAF6 whether at mRNA level or at the protein level. Consistent with above results, the mRNA expression levels of TRAF6 were consistent with the protein expression levels indicating that pre-miR-2187 depresses TRAF6 expression at the posttranscriptional level (Figure 4B). To investigate the regulation of miR-2187 on endogenous TRAF6, we detected the effect of miR-2187 on TRAF6 gene after transfection of MKC cells. Overexpression of miR-2187 significantly inhibited endogenous TRAF6 at protein and mRNA expression level (Figure 4C). In contrast, miR-2187 inhibitors distinctly enhanced TARF6 expression compared with control inhibitors (Figure 4D). In addition, in order to detect whether miR-2187 will inhibit the expression of TRAF6 during viral infection and LPS stimulation. We transfected the miR-2187 into the MKC cells for 48 h, and then stimulated the cells with LPS, SCRV, or poly(I:C). The result (Figure 4E) indicated that the expression of TRAF6 could be significantly enhanced after LPS, SCRV or poly(I:C) treatment, and overexpressed miR-2187 inhibits TRAF6 expression in MKC cells. In contrast to the transfection of miR-2187, miR-2187 inhibitor can significantly enhance the expression of TRAF6 after LPS, SCRV, or poly(I:C) treatment (Figure 4F). Overall, these results indicated that miR-2187 inhibits the expression of TRAF6 gene at posttranscriptional levels, and miR-2187 can also inhibit TRAF6 expression during LPS stimulation or RNA virus infection.

### miR-2187 Depresses TRAF6-Mediated NF-κB and IRF3 Signaling Pathway

**Figure 5:**
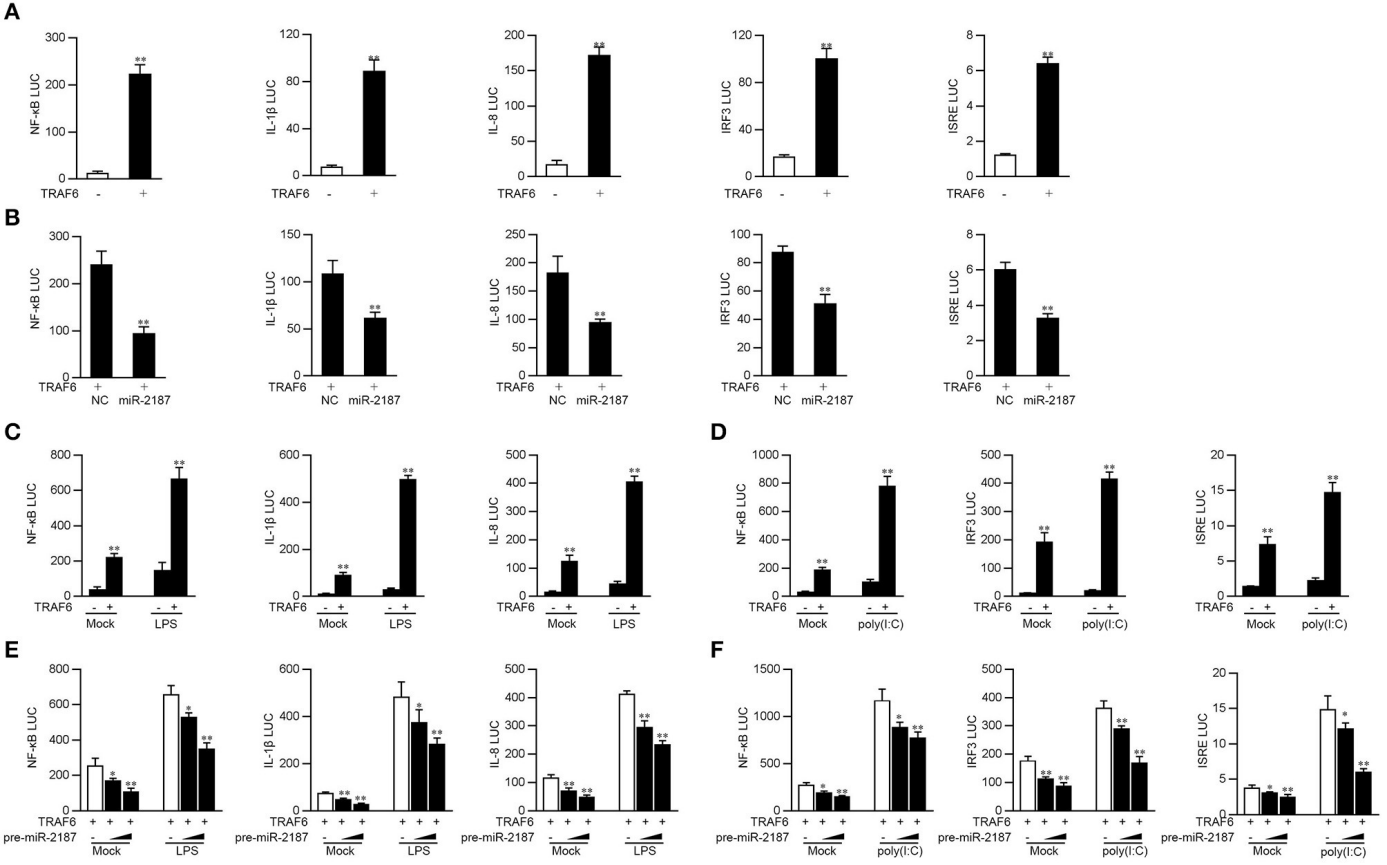
Overexpressed miR-2187 depresses TRAF6-mediated NF-κB and IRF3 signaling. **(A)** Co-transfect EPC cells with TRAF6 expression plasmid, phRL-TK *Renilla* luciferase plasmid and NF-κB, IL-1β, IL-8, IRF3, or ISRE luciferase reporter gene for 24 h, and then measure luciferase activity. **(B)** Luciferase test showed that co-transfection of TRAF6 expression plasmid and NC or miR-2187 with EPC reduced luciferase activity. Co-transfect EPC cells with TRAF6 expression plasmid and stimulated with LPS (1 mg/ml) **(C)** for another 6 h or poly(I:C) (1 mg/ml) for 12 h **(D)**. **(E)** EPC cells were transfected with miR-2187 in a concentration gradient manner, together with luciferase reporter gene for 24 h and stimulated with LPS (1 mg/ml) for another 6 h or poly(I:C) (1 mg/ml) for 12 h **(F)**. Afterward, the luciferase activity was measured. All the luciferase activity was normalized to *Renilla* luciferase activity. All data are presented as the means ± SE from three independent triplicated experiments. **p < 0.01; *p < 0.05 vs. the controls.

Studies have shown that TRAF6 activates the canonical IKK complex, leading to the activation of NF-κB, and can regulate the expression of inflammatory cytokines. Given that miR-2187 can inhibit the production of inflammatory factors and antiviral genes, we explore the underlying regulation mechanism of miR-2187 on TRAF6-induced NF-κB and IRF3 signaling pathway. First, miiuy croaker TRAF6 expression plasmid and NF-κB, IL-1β, IL-8, IRF3, or ISRE reporter genes were co-transfected into EPC cells for 24 h. The results of dual-luciferase reporter assays indicting overexpression of TRAF6 can activate NF-κB and IRF3 luciferase reporter genes, as well as IL-8, IL-1β, and ISRE luciferase reporter genes (Figure 5A). The above results indicated that miR-2187 targets and regulates the TRAF6, we then examined whether overexpression of miR-2187 inhibits the activation of NF-κB, IRF3, and ISRE luciferase reporters by targeting TRAF6. To this end, we transfected with miiuy croaker TRAF6 expression plasmid, together with miR-2187 mimics or negative control mimics into EPC cells. The results showed in Figure 5B, miR-2187 mimics significantly inhibits the activation of NF-κB, IL-1β, IL-8, IRF3, and ISRE induced by the overexpression of TRAF6.

Given that miR-2187 regulates the expression of inflammatory cytokines and antiviral genes, then we tested whether miR-2187 regulates the activity of the indicated luciferase reporter gene under different stimulations. As shown in Figure 5C, although overexpression of TRFA6 markedly up-regulated the luciferase activity of NF-κB, IL-8, and IL-1β reporter genes compared with controls, the activating effects were more significant after stimulation with LPS. Except for LPS stimulation, poly(I:C) stimulation was also used to determine the luciferase activity of antiviral signaling induced by TRAF6. The results showed that the activation trend of IRF3 signal pathway is more obvious than that of the unstimulated control group under overexpression of TRFA6 (Figure 5D). Moreover, the gradient experiment induced by pre-miR-2187 plasmid further verified the indicated results. Pre-miR-2187 showed obviously inhibitory effect on NF-κB, IL-8, IL-1β, IRF3, and ISRE reporter genes during LPS or poly(I:C) infection (Figures 5E,F). Collectively, these data sufficiently demonstrated that miR-2187 could negatively regulate TRAF6-mediated NF-κB and IRF3 signaling pathway upon LPS or poly(I:C) stimulation, respectively.

### Knockdown of TRAF6 Attenuated Antibacterial and Antiviral Immune Response

**Figure 6:**
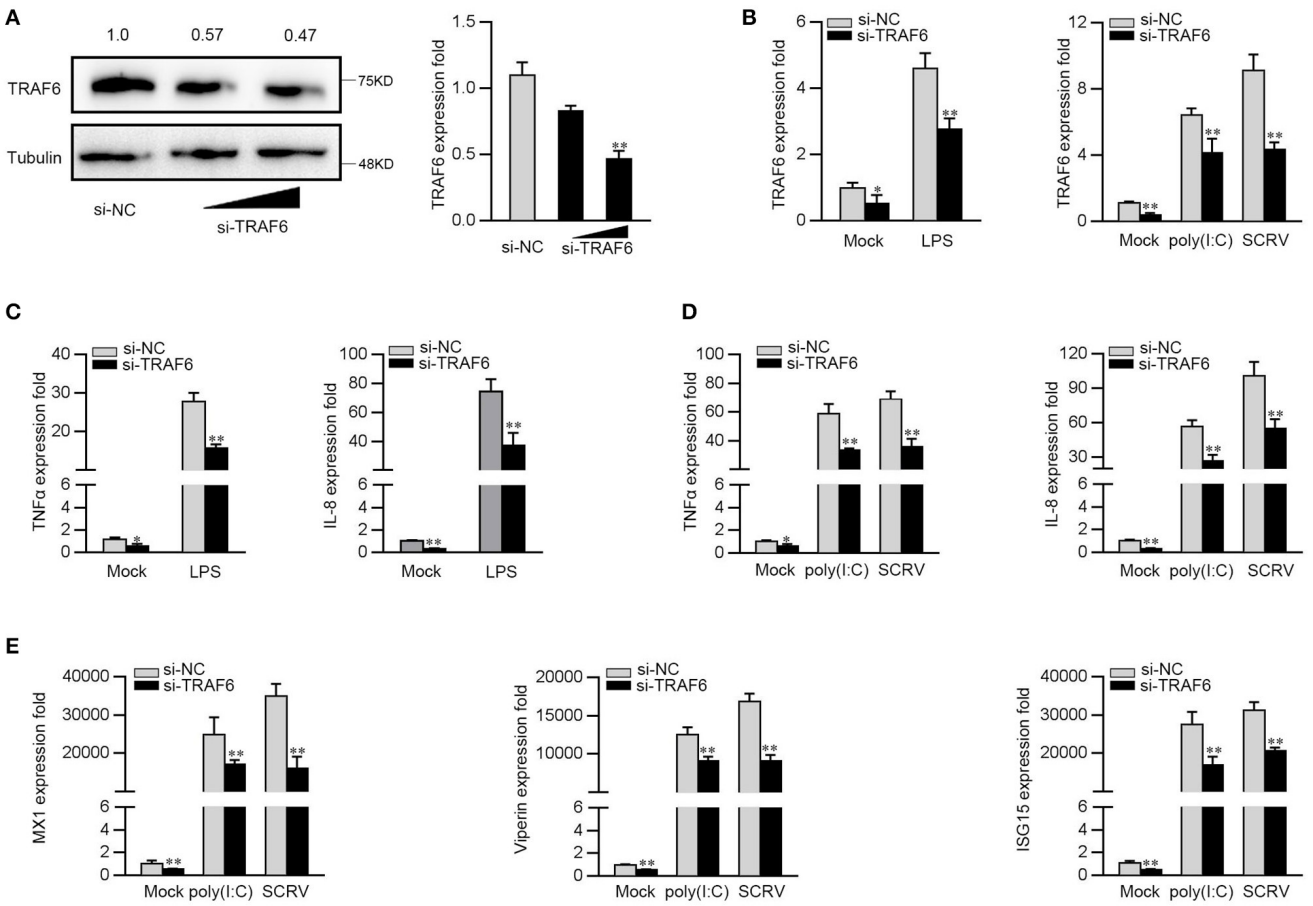
Knockdown of TRAF6 depress the expression of inflammatory factor and antiviral genes. **(A)** MKC were transfected with control siRNA (si-NC) or si-TRAF6 for 48 h, then TRAF6 protein (left panel) and mRNA (right panel) level were determined by western blot and qRT-PCR, respectively. **(B)** MKC were transfected with contrl siRNA (si-NC) or si-TRAF6 for 48 h, the macrophages (MKC) were treated with LPS (left panel), poly(I:C) or SCRV (right panel), respectively. The expression levels of TRAF6 by qRT-PCR. **(C)** MKC cells were transfected with si-NC or si-TRFA6 for 48 h, and treated with LPS for 6 h. The expression levels of TNFα and IL-8 were determined by qRT-PCR. **(D,E)** After transfection of si-NC or si-TRAF6, MKC cells were treated with poly (I:C) or SCRV for another 12 h or 18 h, respectively. The expression levels of TNF-α, IL-8 **(D)**, MX1, Viperin, and ISG15 **(E)**. All data are presented as the means ± SE from three independent triplicated experiments. **p < 0.01; *p < 0.05 vs. the controls.

To further investigate the contribution of TRAF6 to the antiviral and antibacterial response, we silenced TRAF6 to determine the expression of inflammatory cytokines and ISGs upon LPS, poly(I:C) or SCRV treatment. TRAF6-specific small interfering RNA (si-TRAF6) was transfected into MKC cells, the expression levels of endogenous TRFA6 in protein and mRNA were significantly inhibited (Figure 6A). We knocked down TRAF6 and checked the expression of TRAF6 upon the macrophages were treated with LPS, poly(I:C), or SCRV. As shown in Figure 6B, si-TRAF6 effectively suppressed the expression level of TRAF6 in MKC cells after LPS, poly(I:C), or SCRV treatment. Afterwards, MKC cells were transfected with TRAF6-specific siRNA and then stimulated with LPS. As shown in Figure 6C, knockdown of TRAF6 significantly decreased the expression of TNF-α and IL-8 in MKC cells stimulated with LPS. Similar downregulation trends were also detected in MKC cells treated with poly(I:C) or SCRV treatment. As shown in Figures 6D,E, silenced TRAF6 in MKC cells obviously reduces the expression of TNF-α, IL-8, MX1, Viperin, and ISG15. The above results confirmed that miiuy croaker TRAF6 is involved in regulation of antibacterial and antiviral immune responses, and silenced TRFA6 and miR-2187 overexpression have similar effects on TRAF6.

### miR-2187 Feedback Promotes Virus Replication

**Figure 7:**
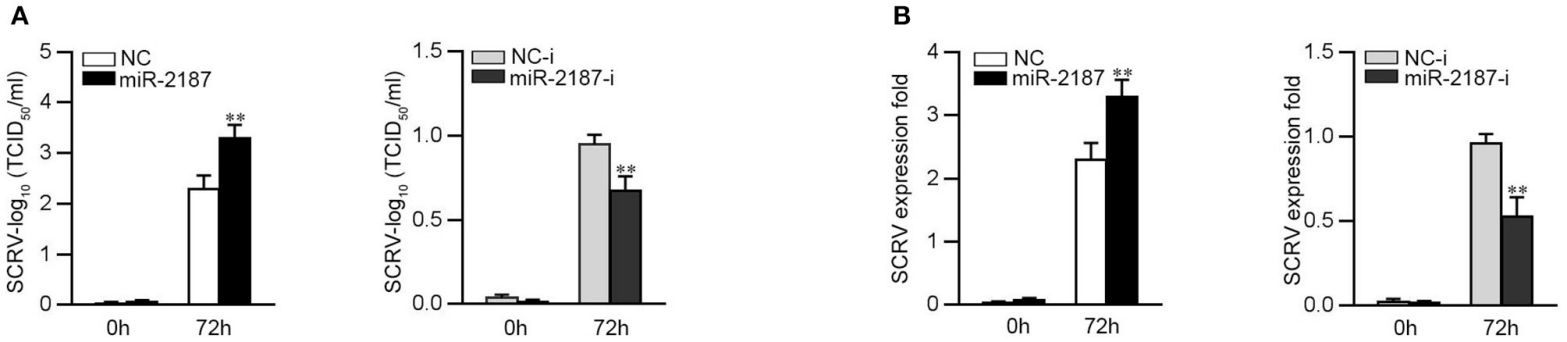
miR-2187 promotes SCRV replication. **(A)** MKC cells were transfected with NC, miR-2187 mimics, NC-i or miR-2187-i, and infected with SCRV at MOI 5 for 1 h and washed and then added with fresh medium. After 72 h, SCRV TCID_50_ in cultural supernatants was measured with EPC cells. **(B)** The qRT-PCR analysis was conducted for intracellular SCRV RNA. All data are presented as the means ± SE from at least three independent triplicated experiments. **p < 0.01 vs. the controls.

To further study the biological significance of the upregulation miR-2187 induced by SCRV, we examined the effect of miR-2187 on SCRV replication in EPC cells. By measuring the SCRV 50% tissue culture infectious dose (TCID_50_) levels in the supernatant from the infected MKC cells, we found that replication of SCRV is facilitated by the overexpression of miR-2187, while inhibition of miR-2187 decreased SCRV replication (Figure 7A). Consistent with these results, overexpression of miR-2187 promotes SCRV replication, while inhibition of miR-2187 attenuated SCRV replication in infected MKC cells (Figure 7B). These results revealed that host miR-2187 can enhance SCRV replication.

### miR-2187 Regulation of TRAF6 Is Widely Found in Teleost Fish

**Figure 8:**
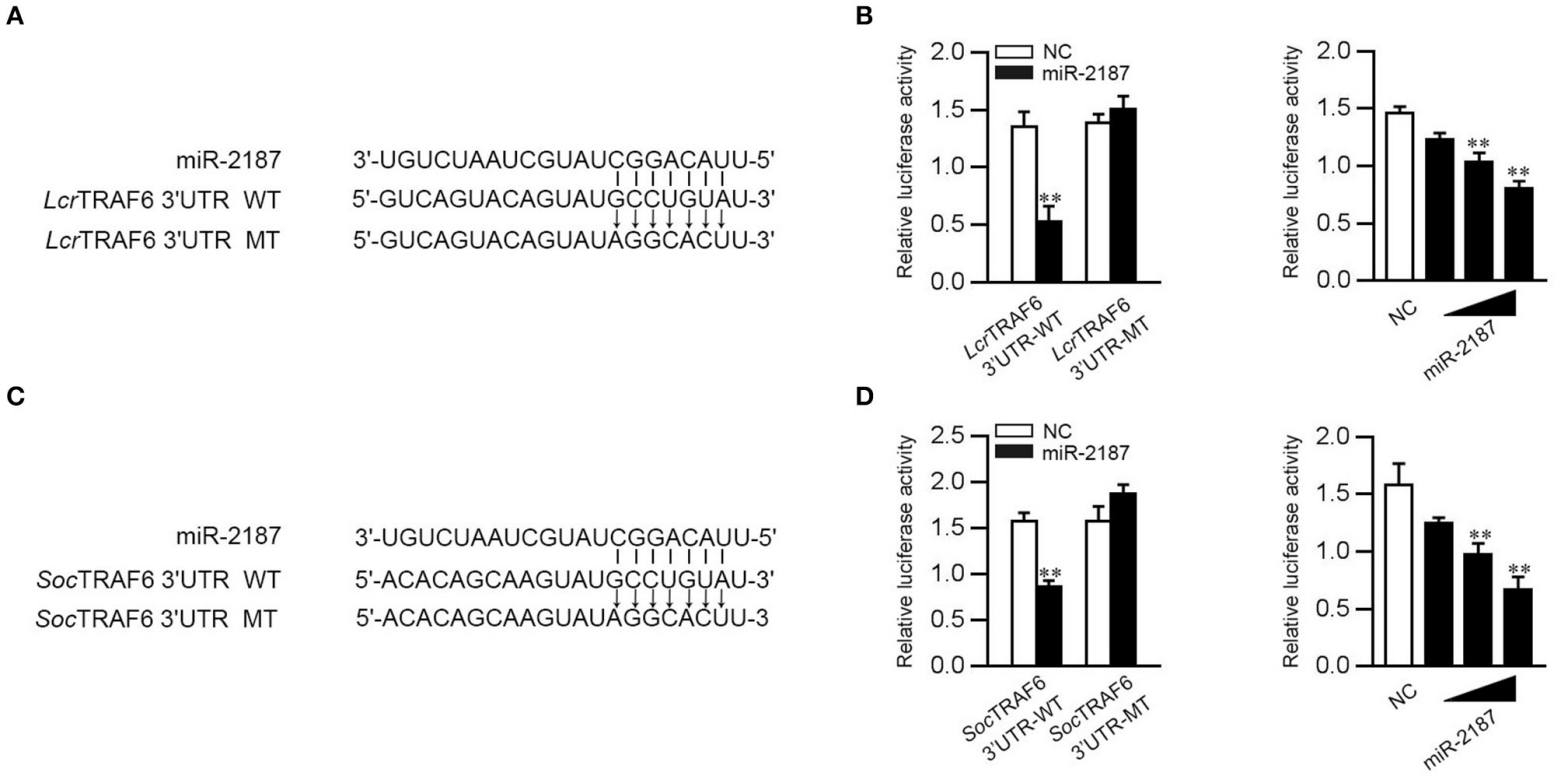
miR-2187 regulation of TRAF6 is widely found in teleost fish. **(A)** Schematic diagram of the predicted target sites of miR-2187 in the 3′UTR of *L. crocea* TRAF6. **(B)** EPC cells were transfected with NC or miR-2187, along with the wild-type or mutant *L. crocea* TRAF6 3′UTR and the luciferase activity was determined (left). The dose gradient experiments were conducted for miR-2187 transfection (right). **(C)** Schematic diagram of the predicted target sites of miR-2187 in 3′-UTR of *S. ocellatus* TRAF6. **(D)** EPC cells were transfected with NC or miR-2187, along with the wild type or the mutant type of *S. ocellatus* TRAF6 3′-UTR and the luciferase activity was determined (left). The dose gradient experiments were conducted for miR-2187 transfection (right). All data are presented as the means ±SE from at least three independent triplicated experiments. **p < 0.01 vs. the controls.

**Figure 9:**
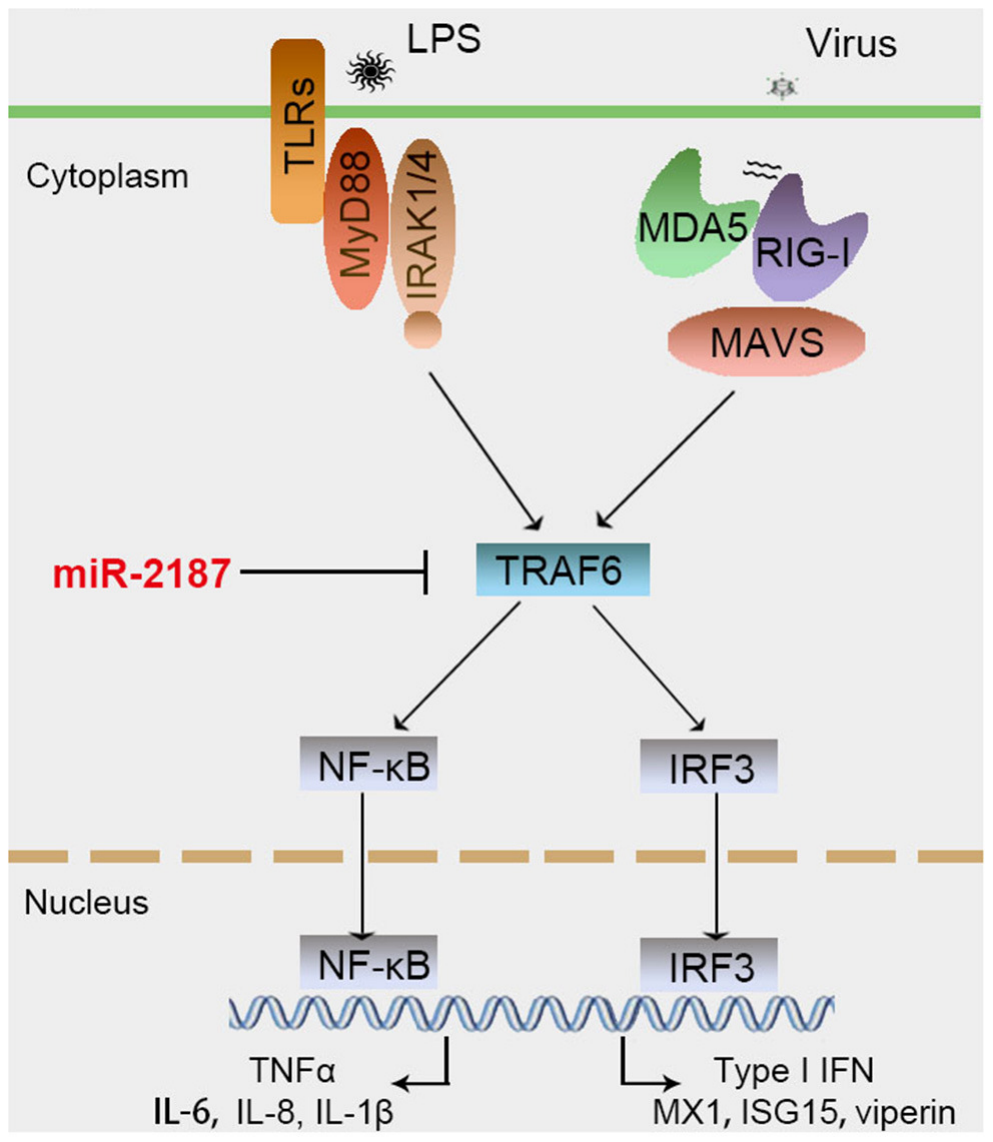
Proposed model for the mechanism by which induced miR-2187 negatively regulates NF-κB and IRF3 signaling expression by targeting TRAF6, thereby avoiding excessive inflammation and immune responses.

To confirm the universality that miR-2187 targets TRAF6, we explored the mechanism in other fish including *L. crocea* and *S. ocellatus*. In this respect, we generated the luciferase reporter constructs by cloning the TRAF6 3′-UTR of *L. crocea* into the pmir-GLO vector within the mutation at the miR-2187 binding site as a control (Figure 8A). miR-2187 mimics significantly suppressed luciferase activity when co-transfected with the *L. crocea* TRAF6-3′UTR reporter plasmid, whereas miR-2187 mimics showed no effect on luciferase activity in cells transfected with mutant types (Figure 8B). We also demonstrated that miR-2187 inhibits luciferase activity in a dose-dependent manner. Meanwhile, we found that miR-2187 has a similar inhibitory effects on luciferase activity when co-transfected with the TRAF6 3′-UTR of *S. ocellatus* (Figures 8C,D). These results indicate that miR-2187 can target the TRAF6 gene in other teleost fish, verifying that the functions of miR-2187 are conserved to some extent. To summarize the above results, these results showed that Gram-negative bacterial or RNA virus infection can enhance the expression of miR-2187. Up-regulated miR-2187 inhibits the production of inflammatory cytokines and antiviral genes by targeting TRAF6 and subsequently inhibiting NF-κB and IRF3 signaling, thereby avoiding excessive inflammation (Figure 9).

## Discussion

Aquaculture has developed rapidly over the past three decades to become an efficient protein producer. However, its development has been hampered by pathogenic infections. Rhabdoviruses are a group of enveloped, single-stranded, negative RNA viruses such as SCRV that can cause severe hemorrhagic septicemia in freshwater and marine fish (29, 38). In addition, *V. anguillarum* is a kind of Gram-negative bacterium of curved rod type, which causes high mortality and severe epidemic vibriosis in many marine animals, accompanied by symptoms of hemorrhagic sepsis (39, 40). Hence, it is urgent matter to explore potential immunomodulatory mechanism of teleost. Teleost use PRRs to identify the invasion of pathogenic microorganisms. Similar to mammals, the PRRs of teleost initiate immune response by recognizing the conservative sequence of PAMPs, and then signal the transcription of NF-κB and IRF3/7, leading to the production of inflammatory cytokines and antiviral genes, producing a clearance effect on pathogen invasion (41–43). However, the excessive activation of TLR or RIG-I signals can disrupt the immune balance and cause self-diseases.

As a member of the RLRs family, RIG-I has been widely studied for its role in detecting viral RNA and antiviral immune response. After activated RIG-I, NF-κB /IRF3 will transfer from the cytoplasm to the nucleus, and induce the transcription of multiple innate immune genes. MAVS is a key downstream adaptor of RIG-I which contains multiple TRAF interaction motifs in the proline-rich region, which are related to the TRAF family (44, 45). Specifically, TRAF6 is a member of the TRAF family. TRAF6 signaling activates the IKK complex, leading to the activation of NF-κB and the expression of inflammatory cytokines, thereby eliminating the invasion of the virus (46). At the same time, the IRF family is also directly activated by TBK1/IKKε. In the innate immune response, from invertebrates to mammals, the signal transduction of the TLR pathway is highly conserved. Toll-like receptors detect pathogen-related molecular patterns (PAMPs) and initiate signaling pathways to resist the invasion of pathogens. When the TLR binds to the corresponding ligand, the IL-1 receptor-associated kinase 4 (IRAK4) will recruit MyD88, further activate TRAF6, and ultimately lead to the activation of NF-κB (47). As indispensable upstream mediators of the NF-κB pathway, TRAF6 has been reported to be regulated by many different molecules. For example, the heat shock protein 27 (HSP27) binds to TRAF6 to regulate the signaling pathway induced by IL-1 (48). The deubiquitinating enzymes such as cylindromatosis (CYLD) can inhibit the activation of MyD88 and TRIF-dependent NF-κB by deubiquitinating TRAF6 (49). Further investigations have reported that overexpression of miR-146a attenuates POCD hippocampal-dependent learning and memory impairment by targeting IRAK1 and TRAF6 (50). In this study, we found that TRAF6 enhances inflammatory cytokine production *via* modulating NF-κB signaling in miiuy croaker. Furthermore, we demonstrated that overexpression of fish TRAF6 could induce the activation of IRF3 signaling and several IFN-inducible genes, such as MX1, Viperin, and ISG15, which suggests the significant role of TRAF6 in host antiviral immunity. However, the regulation of NF-κB and IRF3 signaling pathway by miRNA targeting TRAF6 in fish has not been studied in depth.

Due to the pivotal regulatory role of miRNA, research on the mechanism of miRNA regulating immune response has also been paid more and more attention. At present, miRNAs functions as fine-tuning regulators involved in various biological processes of fish has been increasingly clarified. In *Epinephelus tauvina*, miR-1, miR-30b, miR-150, and miR-184 all function as important regulatory factor upon neuronecrosis virus infection (44). Meanwhile, such as miR-146a (51) and miR-98 (52) have been shown to be involved in innate and adaptive immune responses in teleost fish. As a key regulator of immune response, miRNA not only response to microorganism invasion, but also regulates the replication of some pathogens. For example, miR-30c can be upregulated by porcine reproductive and respiratory syndrome virus, and inducible miR-30c inhibits IFN-I signaling by targeting JAK1, thereby promoting viral replication (53). In this study, we found for the first time that the expression of miR-2187 was rapidly upregulated in miiuy croaker after stimulation with Gram-negative bacteria and RNA virus. Upregulated miR-2187 could suppress the production of inflammatory cytokines and antiviral genes. Additionally, overexpression of miR-2187 could promote the publication of SCRV. Overall, these results indicated that inducible miR-2187 decreases the production of inflammatory cytokines and ISGs in LPS- and poly(I:C)-stimulated macrophages through negatively regulating the NF-κB and IRF3 signaling pathway by modulating TRAF6. This could be a novel discovery about the negative regulation of the antibacterial and antiviral immunity, which also enriches the network of miRNAs-induced immune response mechanism in teleost fish.

In conclusion, this study demonstrated that host miR-2187 as a new regulator of TRAF6 plays a negative regulatory role in TRAF6-mediated NF-κB and IRF3 signaling pathways, which provide direct evidence for the role of miR-2187 in antiviral and antibacterial response. In addition, TRAF6 as a new target of miR-2187 has also been confirmed in other teleost fish, proving that miR-2187 targeting TRAF6 is conserved in other fishes. Overall, our research not only enables us to increase our understanding of miR-2187 function, but also provides insight into the regulatory network of miRNA-mediated immune responses against fish pathogen infections.

## Data Availability Statement

The original contributions presented in the study are included in the article/Supplementary Material, further inquiries can be directed to the corresponding author/s.

## Ethics Statement

The animal study was reviewed and approved by Research Ethics Committee of Shanghai Ocean University.

## Author Contributions

TX: conceived and designed the experiments. WG and RC: performed the experiments and analyzed the data. YS and TX: contributed reagents, materials, and analysis tools. WG and TX: wrote the paper. All authors contributed to the article and approved the submitted version.

## Conflict of Interest

The authors declare that the research was conducted in the absence of any commercial or financial relationships that could be construed as a potential conflict of interest.
