# Supplementary material for: MicroRNA-2187 Modulates the NF-κB and IRF3 Pathway in Teleost Fish by Targeting TRAF6
Source: Front Immunol. 2021 Feb 15;12:647202. doi: 10.3389/fimmu.2021.647202 (PMC7917119; doi:10.3389/fimmu.2021.647202)
Supplement: Supplementary file 1 [file Table_1.DOCX]

**Supplementary Table 1** PCR primer sequence information in this study

| **Primers** | **Sequences (5’-3’)** | **Application** |
| --- | --- | --- |
| **Real-time PCR** | | |
| TRAF6-RT-1F | ATGATGGAAAAGGAACGGGAAT | Expression of TRAF6 |
| TRAF6-RT-1R | TCGGACAGCGAACAGTTAGTGA |  |
| IL-8-RT-1F | AGCAGCAGAGTCTTCGT | Expression of IL-8 |
| IL-8-RT-1R | TCTTCGCAGTGGGAGTT |  |
| TNF-α-RT-1F | GTTTGCTTGGTACTGGAATGG | Expression of TNF-α |
| TNF-α-RT-1R | TGTGGGATGATGATCTGGTTG |  |
| IL-1β-RT-1F | CATAAGGATGGGGACAACGAG | Expression of IL-1β |
| IL-1β-RT-1R | TAGGGGACGGACACAAGGGTA |  |
| Viperin-RT-1F | ACCCGTCCAAGTCCATAC | Expression of Viperin |
| Viperin-RT-1R | TCATGTCAGCTTTGCTCC |  |
| MX1-RT-1F | GCTGCTTGTTTACTCCCA | Expression of MX1 |
| MX1-RT-1R | ACCTGCATCATCTCCCTC |  |
| ISG15-RT-1F | TGAACGGACACAAGACGC | Expression of ISG15 |
| ISG15-RT-1R | TGAGGAATACCTGCATGG |  |
| SCRV-RT-1F | CTCCTTCTGCGGATGCTA | Expression of SCRV |
| SCRV-RT-1R | TCGGGATTTGCTCTACCAG |  |
| β-actin-RT-1F | GTGATGAAGCCCAGAGCA | Expression of β-actin |
| β-actin-RT-1R | CGACCAGAGGCATACAGG |  |
| miR-2187-RT-1F | CGCAGTTACAGGCTATGCT | Expression of miR-2187 |
| miR-2187-RT-1R | GGTCCAGTTTTTTTTTTTTTTTACAGA |  |
| 5.8S rRNA-RT-1F | AACTCTTAGCGGTGGATCA | Expression of 5.8S rRNA |
| 5.8S rRNA-RT-1R | GTTTTTTTTTTTTTTTGCCGAGTG |  |
| **Vector construction** | | |
| pre-miR-2187-1F | CCCAAGCTTTATTGTATTACGGTAGGATG | Amplification of pre-miR-2187 |
| pre-miR-2187-1R | CCGGAATTCCAGATAGCACGAACTTTT |  |
| TRAF6-3’UTR-1F | CGAGCTCTAAACTCTACTTATCCGCTGTG | Amplification of TRAF6-3’UTR |
| TRAF6-3’UTR-1R | CTAGTCTAGACTGCTTATTGTTTCACCC |  |
| TRAF6-3’UTR-GFP-1F | CCCAAGCTTGCTAGACTCTACTTATCCGCTGTG | Amplification of TRAF6-3’UTR |
| TRAF6-3’UTR-GFP-1R | CGCGTCGACCTGCTTATTGTTTCACCC |  |
| TRAF6-1F | GACGATGACGACAAGAAGCTTATGGCTTGCATTGACAGCAAT | Amplification of the CDS and 3’-UTR of TRAF6 |
| TRAF6-1R | TCCTTGCATGGTGTGGACAAAGAGGGAGA |  |
| TRAF6-3’UTR-WT-1F | TTGTCCACACCATGCAAGGAGCTTTTGACGGG |  |
| TRAF6-3’UTR-WT-1R | TGATGGATATCTGCAGAATTCTTGCTGATTCAACCTTTCTGCA |  |
| TRAF6-3’UTR-MT-1F | CAGCAAGTATaggcactTTTATGTCGATGTCAATGGGACTT | Mutation of TRAF6-3’UTR |
| TRAF6-3’UTR-MT-1R | agtgcctATACTTGCTGTGTCCAGACTTAAAATG |  |
| *Lcr*-TRAF6-3’UTR-WT-1F | CGCGAGCTCCTCGGTTTCAAGGGACTA | Amplification of *L. crocea* TRAF6-3’UTR |
| *Lcr*-TRAF6-3’UTR-WT-1R | TGCTCTAGAGGCTGATTCAACCTTCTG |  |
| *Lcr*-TRAF6-3’UTR -MT-1F | GTATGCaggcactTTTATGTCGACGTCAATGGGATT | Mutation of *L. crocea* TRAF6-3’UTR |
| *Lcr*-TRAF6-3’UTR-MT-1R | CATAAAagtgcctGCATACTGTACTGACATCTCAGCATG |  |
| *Soc*-TRAF6-3’UTR-WT-1F | CGAGCTCCTGATGGACACCAGAAGG | Amplification of *S. ocellatus* TRAF6-3’UTR |
| *Soc*-TRAF6-3’UTR-WT-1R | CCCTCGAGGGCTTTGACTGCTAGTGG |  |
| *Soc*-TRAF6-3’UTR-MT-1F | GTATGCaggcactTTTATGTCAATGTCAATGGGACTTC | Mutation of *S. ocellatus* TRAF6-3’UTR |
| *Soc*-TRAF6-3’UTR-MT-1R | CATAAAagtgcctGCATACTTGCTGTGTCCAGACTTAA |  |
